# Optimization of FDM 3D printing process parameters to produce haemodialysis curcumin-loaded vascular grafts

**DOI:** 10.1007/s13346-021-01078-2

**Published:** 2021-10-12

**Authors:** Sara Basile, Essyrose Mathew, Ida Genta, Bice Conti, Rossella Dorati, Dimitrios A. Lamprou

**Affiliations:** 1 School of Pharmacy, Queen’s University Belfast, 97 Lisburn Road, Belfast, BT9 7BL UK; 2 Department of Drug Sciences, University of Pavia, Viale Taramelli 12, 27100 Pavia, Italy

**Keywords:** 3D printing, Hot-melt extrusion, Haemodialysis, Fistula, Drug delivery, Curcumin

## Abstract

3D printing has provided a new prospective in the manufacturing of personalized medical implants, including fistulas for haemodialysis (HD). In the current study, an optimized fused modelling deposition (FDM) 3D printing method has been validated, for the first time, to obtain cylindrical shaped fistulas. Printing parameters were evaluated for the manufacturing of fistulas using blank and 0.25% curcumin-loaded filaments that were produced by hot melt extrusion (HME). Four different fistula types have been designed and characterized using a variety of physicochemical characterization methods. Each design was printed three times to demonstrate printing process accuracy considering outer and inner diameter, wall thickness, width, and length. A thermoplastic polyurethane (TPU) biocompatible elastomer was chosen, showing good mechanical properties with a high elastic modulus and maximum elongation, as well as stability at high temperatures with less than 0.8% of degradation at the range between 25 and 250 °C. Curcumin release profile has been evaluated in a saline buffer, obtaining a low release (12%) and demonstrating drug could continue release for a longer period, and for as long as grafts should remain in patient body. Possibility to produce drug-loaded grafts using one-step method as well as 3D printing process and TPU filaments containing curcumin printability has been demonstrated.

**Graphical abstract:**

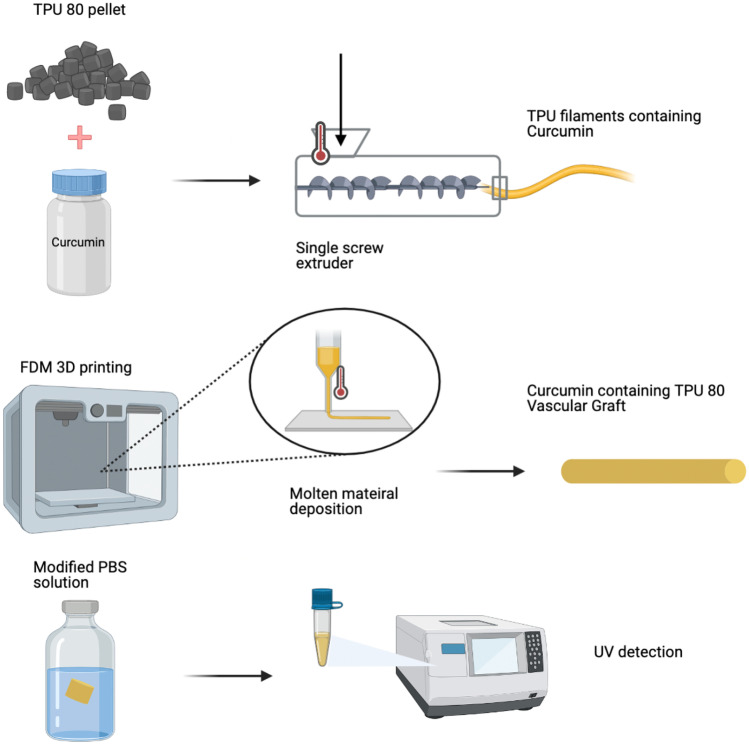

## Introduction

Chronic kidney disease (CKD) is a pathologic condition characterized by the decreased function and altered structure of the kidneys. The kidneys have a variety of functions, including filtering the blood to eliminate waste and excess fluid, to support red blood cells and to help controlling the blood pressure [1]. In 2017, there were 697.5 million cases of CKD in the world, with 9.1% of prevalence and 1.2 million deaths [2]. According to the World Health Organization (WHO), from 2000 to 2019, kidney diseases have risen from the world’s 13th leading cause of death to the 10th. Mortality has increased from 813,000 in 2000 to 1.3 million in 2019 [3].

When impaired kidneys are functional only at 10–15%, patients undergo a dialysis treatment for replacing healthy kidneys [1]. Haemodialysis (HD) is a technique where blood circulates through dialyser membrane channels, while the dialysis fluid with an electrolyte composition like extracellular fluid is passing outside these channels in the opposite direction, in order to eliminate wastes from the patient blood [4].

Entrance points obtained with a non-invasive surgical procedure are required into patient blood vessels for patients under HD to connect blood to the dialyser [1]. A well-functioning vascular access (VA) will lead to an efficient HD procedure. Native arteriovenous fistula (AVF) is the first preferred approach, arteriovenous graft (AVG) and central venous catheter being the main types of dialysis access used [5]. AVG is an AVF that has been made with prosthetic interposition between an artery and a vein to link two vessels, which could not be connected by AVF due to the distance between them [5]. AVF remains the first choice for haemodialysis access, compared to AVG and catheter; however, when AVF become unappropriated due to unsuitable veins or maturation issues, AVG is the second choice to mitigate catheter use, mostly in elderly population [6].

A variety of synthetic vascular grafts were made in the past decade that composed of polytetrafluoroethylene (ePTFE) or polyethylene terephthalate (Dacron^©^), which can successfully apply to replace large diameter vessels (> 6.0 mm). These products show some issues that are related to the replacement of small diameter blood vessels and to their susceptibility to infection [7]. Furthermore, grafts could need to be placed in a looped configuration; if the loop is too tight, it could lead to kinking at the apex and reducing diameter dimension [8]. Therefore, the aim is to fabricate grafts according to the vasculature of each patient. Tissue-engineering technologies could apply for overcoming commercial-related graft problems considering possibility nowadays to obtain bio-mimicking vascular tubes [9].

In 1960s, xenogeneic, and later allogeneic, grafts are used which did not lead to immunogenic reaction after implantation; however, they needed biological treatment to remove collagen, connective tissue proteins and cellular remnants, with the aim of decrease in immunogenicity [10]. Furthermore, cryopreserved grafts or femoral vein homograft results failed in clinical environments and are rarely used, even if they are available in commerce [11].

Electrospinning is a well-established technique for the fabrication of small diameter cell-free scaffold tissue-engineered vascular grafts (TEVGs), with adjustable nano- and micro-fibres, porous structures favourable for cell infiltration and regeneration [12]. Electrospinning parameters, such as electric field, distance needle collector, flow rate and needle diameter, and solution parameters including solvent, polymer concentration, viscosity and solution conductivity and environmental parameters (humidity, temperature) could affect electrospun fibre characteristics (e.g. smooth, bead-free) [13].

3D printing (3DP) is a new prospective in the medical technologies’ era, to produce TEVGs, due to the possibility to print structures composed of cells, growth factors, drugs and scaffolds assembled in an organized way [14]. 3DP systems have shown a promising solution compared to the electrospinning inability to control a three-dimensional structure [15]. In addition, 3DP could reduce fabrication time and costs, as both cells and scaffolds can be processed at the same time [14].

One of the recurring issues using 3DP technologies is that often a scaffold is used to maintain layers position for achieving the requested structure, such as cylindrical shape [16]. However, 3DP method could be a potential application for creating patient characteristics that matched vascular grafts, and the versatility of using different types of materials during printing process, providing the possibility to add drugs to the final device [17]. In vascular graft manufacturing, anti-coagulant drugs could be added to reduce thrombosis events, which is one of the main vascular graft related consequences. In the last 4 years, the use of 3DP has been one of the latest prospects in this field; however, only a few studies are present in the literature regarding vessel constructs or vascular grafts [8].

For the successful manufacturing of grafts, printing adjustments are required due to the specific vessel shape, such as the rotary system to obtain cylindrical geometries and the avoidance on using support materials for printing a cell-laden bioink [18]. Different materials could be used in order to obtain biocompatible vascular grafts [19], such as sodium alginate, 4-arm-poly(-ethylene glycol)-tetra-acrylate (PEGTA) and gelatin methacryloyl (GelMA), using an inkject bioprinter with coaxial nozzles [20]. Bioinks based on hydrophilic polymer, such as 4-acryloylmorpholine (ACMO), have been employed for 3D printed grafts using a digital light processing (DLP) technology [8].

In this work, vascular grafts were produced using 3DP by adjusting different parameters to find a suitable method to print required shapes. Required cylindrical shapes are hard to obtain using a 3DP method, mainly due to material characteristics, 3D printer working methods and shape geometry. However, the fused deposition modelling (FDM) 3DP has been demonstrated, in our studies, to be suitable to produce vascular grafts with small size diameter (< 6.0 mm). FDM technology provides an accurate pore dimension, morphology and interconnectivity of grafts architectures control, while giving important benefits such as price, high speed and simplicity [21]. After testing different TPU grades (TPU70, TPU80 and TPU95), TPU 80 was the selected polymer, which has been generally used for biomedical applications [22], such as catheter production [17]. Curcumin has been chosen in this study to be added consequently due to its anticoagulant [23, 24] and anti-bacterial properties [25], in order to overcome graft related problems mentioned above. Hot melt extrusion (HME) was used to combine TPU 80 with curcumin to create 0.25% drug-loaded filaments, which could then be used to print drug-loaded vascular grafts. The grafts were then characterized using a variety of physicochemical characterization techniques.

## Materials and methods

### Materials

Elastollan thermoplastic polyurethane (TPU) pellets were kindly provided by DistruPol Ltd. (Dublin, Ireland). Curcumin was purchased from Tokyo Chemical Industry Ltd (UK), the Castor oil was obtained from Ransom (Hitchin, UK) and the phosphate buffer solution (PBS) at pH 7.4 was prepared using PBS tablets, Merck (Darmstadt, Germany). Methanol, ethanol and Tween 80 were purchased from Merck (Darmstadt, Germany), all in analytical grade.

#### CAD

**Fig. 1:**
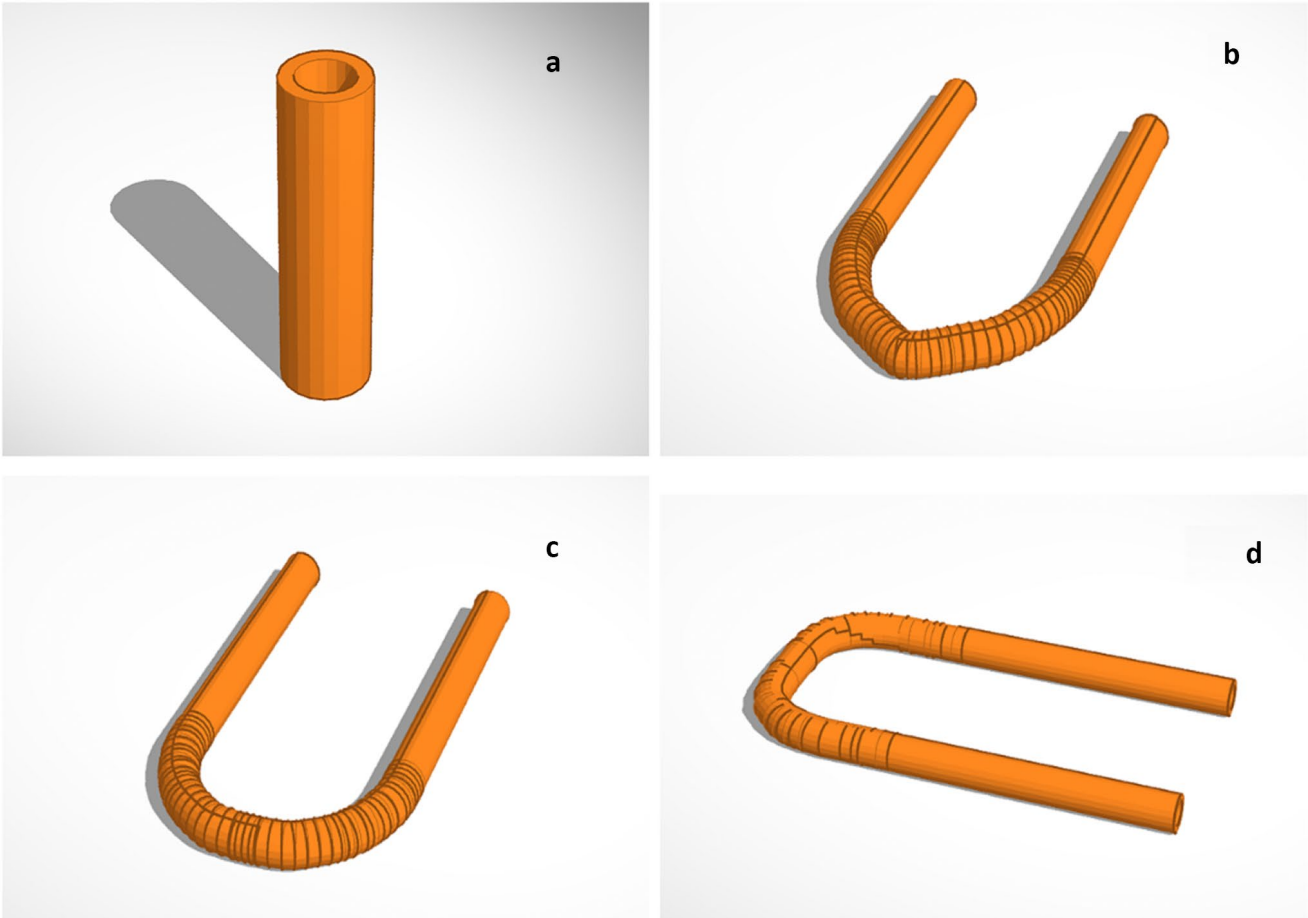
Vascular grafts at different shapes: straight (**a**), V-shape (**b**), U-shape (**c**) and curves-shape (**d**)

Using a computer design system (*Thinkercad*^*©*^), four different vascular grafts were designed (Fig. 1; Table 1), to avoid kinking problems related to straight shape.

**Table 1:** Designed CAD dimensions

**Design**	**Outer diameter (mm)**	**Inner diameter (mm)**	**Wall thickness (mm)**	**Length and width (mm)**
**Straight**	6.0	4.0	2.0	60.0
**V-shape**	6.0	4.0	2.0	41.6
**U-shape**	6.0	4.0	2.0	40.0
**C-shape**	6.0	4.0	2.0	40.5

### Preparation of vascular grafts with TPUs using FDM 3D printer

TPUs vascular grafts were prepared exploiting the FDM 3DP system. Three different TPU elasticity grades (95A, 80A, 70A) were investigated to create vascular grafts (Table 1) using an Ultimaker 2 (Ultimaker B.V., Geldermalsen, the Netherlands) fused filament fabrication (FFF) system and Cura software. The Ultimaker 2 FFF system was equipped with a 0.4-mm nozzle, which is a RepRap Open Source FFF equipment. Different optimized printing parameters are used for the three TPUs (e.g. 95, 80, 70) due to their different characteristics (Table 2).

**Table 2:** 3D printing optimized parameters for TPU 95, 80 and 70

**TPU type**	**Print-head T (°C)**	**Bed T (°C)**	**Layer height (mm)**	**Speed mm s**^−**1**^	**Infill density (%)**
**95**	235	70	0.2	5.0	100
**80**	210	60	0.1	2.0	100
**70**	210	60	0.2	2.0	100

### Preparation of TPU filaments containing curcumin and vascular grafts

TPU filaments containing curcumin were prepared using a single-screw HME. Oil method using castor oil was used to ensure curcumin coating on TPU pellet surface. After filling a 50-mL tube with TPU 80 pellets (30 g), castor oil (30 μL) was added into the tube, and the solution was vortexed to achieve a homogeneous coating of pellets. Oil-coated pellets were then transferred to a new 50-mL tube to avoid loss of drug, by sticking to leftover oil on the tube walls. After that, 0.25% of curcumin was added and vortexed to coat all pellets. Coated pellets were added to filament extruder (3Devo, Utretch, the Netherlands) at an extrusion speed of 3 rpm and a filament fan speed of 80%. The temperature was adjusted according to the material between 170 and 200° C and filament with 2.85 mm diameter extruded.

### 3D printed vascular graft characterization

#### Microscopy

Filaments and 3D printed grafts were assessed using a Leica EZ4 W digital microscope (Leica, Wetzlar, Germany). The surface morphology of the 3D printed grafts with and without curcumin was also evaluated using scanning electron microscope (SEM) (Hitachi TM3030, Tokyo, Japan).

#### FT-IR spectroscopy

Pellet TPU80, curcumin and TPU filaments containing curcumin were analysed by attenuated total reflection-Fourier transform infrared spectroscopy (ATR-FTIR) to establish if any interactions were present between drug and TPU. Spectra were recorded using a Nicolet™ iS50 FTIR Spectrometer (Thermo Fisher Scientific, UK).

#### Thermal analysis

Thermal degradation behaviour was investigated due to high temperatures required during HME and 3DP. A Q500 thermogravimetric analyser (TGA, TA Instruments, New Castle, DE, USA) was used to perform TGA on 5–10 mg TPU80, curcumin and TPU filaments containing curcumin, to measure their weight loss. The analyses performed from 25 to 250 °C with a 10 °C increase per minute and a nitrogen flow rate of 40 mL min^−1^.

Moreover, differential scanning calorimetry (DSC) was also performed to determinate material transitions subjected to high temperatures. DSC 214 Polyma for Polymer Characterization from Netzsch was used, with a range temperature from 25 to 250 °C with a 10 °C increase per minute and a nitrogen flow rate of 40 to 60 mL min^−1^.

### Mechanical properties

TA.XT plus texture analyser (Stable Micro Systems, Surrey, UK) was used to evaluate the elastic modulus and the fracture force of the TPU 80 and filaments containing curcumin. Pieces of filament (6.0 cm) were cut and clamped vertically into the texture analyser, where the distance between both clamps was 2.0 cm and a test speed of 3.0 mm s^−1^. The elastic modulus was calculated from the slope from obtained stress/strain curves.

### In vitro drug release studies

Drug release study was performed to calculate the amount of curcumin eluting from vascular grafts. Due to curcumin low solubility, vascular grafts were placed in 3 mL of PBS solution containing 0.5% v/v Tween 80 [26–28]. PBS with 0.5% Tween 80 and 3% methanol (PTM) was placed in vial placed in a shaking incubator at 37 °C and 40 rpm, according to literature [29]. Different intervals were chosen to analyse the samples (e.g. 2, 4, 6, 24, 48, and 72 h, 1 week and 2 weeks). At each time point, grafts were removed from previous PTM, dried and placed in a fresh 3 mL PTM.

### Degradation study

Degradation study took place using gravimetric analysis by placing 1 cm vascular graft in PBS (7.2 pH) and in PTM at 37 °C and were performed at 24, 48, 72 h, 1, 2 weeks and 3 weeks, to establish TPU filaments containing curcumin and TPU 80 mass loss into the two buffers.

### Statistical analysis

Quantitative data was expressed as a mean ± standard deviation, *n* = 3. The statistical analysis was performed using a one-way analysis of variance; *p* < 0.05 was considered statistically significant.

## Results and discussions

### Fabrication of vascular grafts

**Fig. 2:**
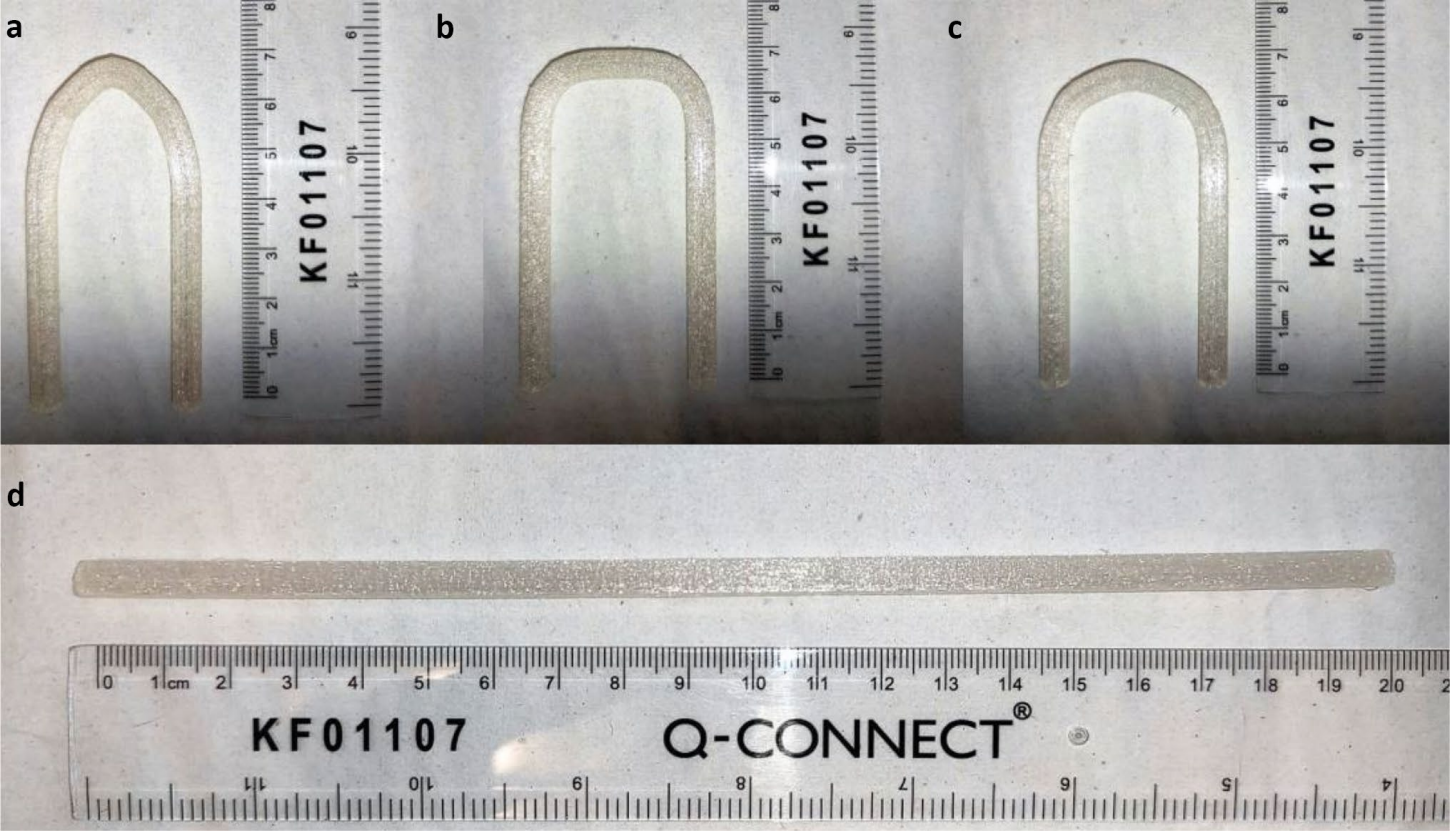
3D printed vascular grafts: V-shape (**a**), C-shape (**b**), U-shape (**c**), and straight (**d**)

The FDM 3DP system has been used to print a commercial TPU 95A (Ninjaflex) filament, to optimize the process printing parameters using three samples for each design and to demonstrate the possibility printing the required shapes with high grades of accuracy. Several parameters needed to be adjusted to print TPU 95; printing process has been performed at different speeds decreasing to 5.0 mm s^−1^ as optimized speed and 0.2 mm layer height, with 100% infill. Three samples for each design were printed with an outer diameter (OD) of 6.0 mm, an inner diameter (ID) of 4.0 mm and a wall thickness of 2.0 mm (Fig. 2). Obtained samples have been measured using light microscopy or a calliper, as shown in Table 3. The most significance differences are related to wall thickness, mostly in V-shape, U-shape and C-shape, due to difficulties of printing these specific shapes. Straight sizes are more closed than other designed shapes/sizes. However, size differences between designed and obtained grafts were included within the statistically accepted values; consequently, printing process accuracy has been demonstrated.

**Table 3:** Comparison of the designed and printed values

**Graft**	**Outer diameter (mm)**		**Inner diameter (mm)**		**Wall thickness (mm)**		**Width and length (mm)**	
	*Designed*	*Obtained*	*Designed*	*Obtained*	*Designed*	*Obtained*	*Designed*	*Obtained*
**Straight**	6.0	5.63 ± 0.64	4.0	3.90 ± 0.01	2.0	0.95 ± 0.61	60.0 (l)	59.31 ± 0.68
**V-shape**	6.0	5.84 ± 0.14	4.0	3.61 ± 0.37	2.0	1.07 ± 0.92	41.56 (w)	40.78 ± 1.69
**U-shape**	6.0	5.92 ± 0.08	4.0	3.51 ± 0.48	2.0	1.10 ± 0.89	40.0 (w)	39.46 ± 0.59
**C-shape**	6.0	5.87 ± 0.12	4.0	3.89 ± 0.17	2.0	1.02 ± 0.97	40.50 (w)	39.47 ± 1.02

**Fig. 3:**
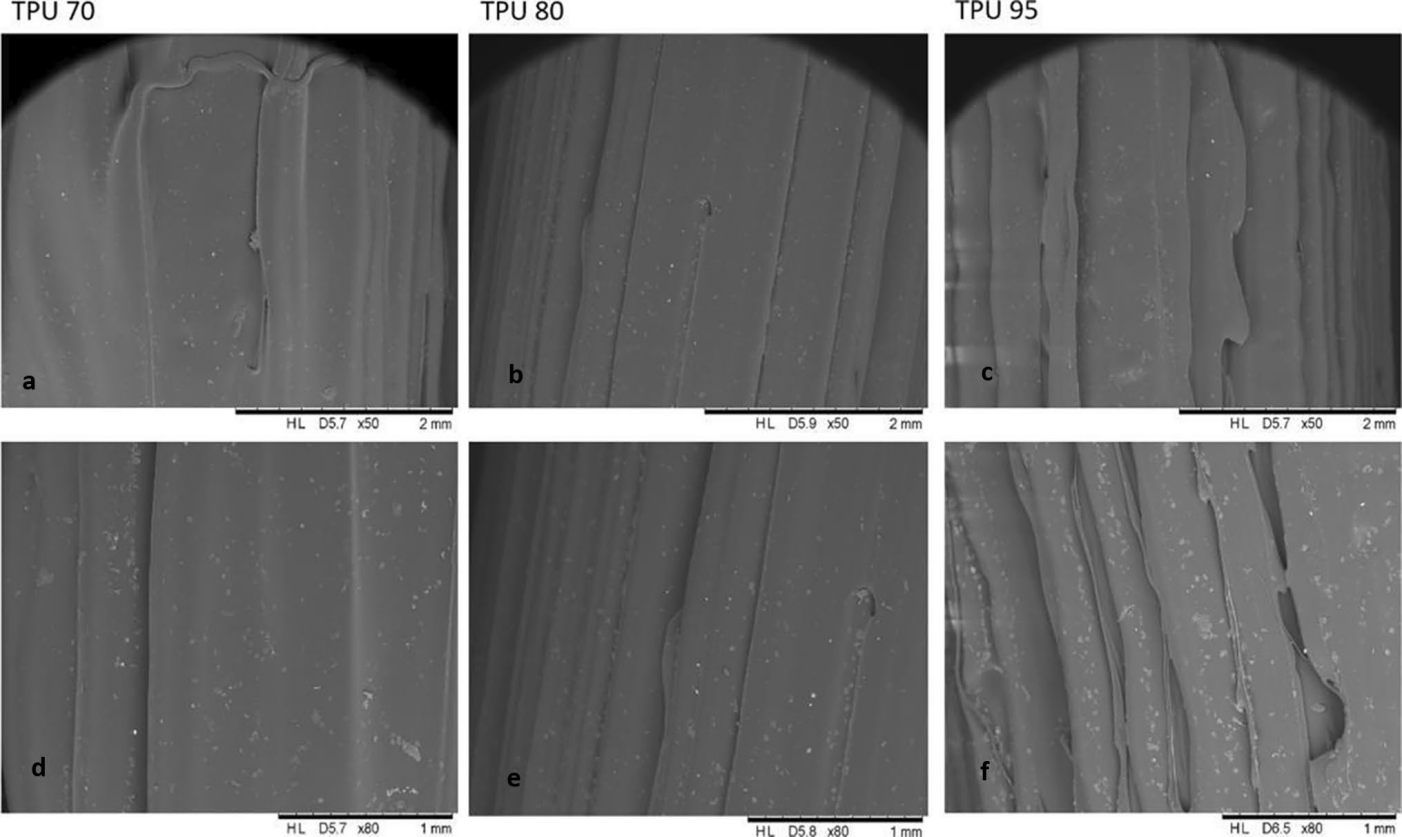
SEM images of 3D printed vascular grafts using TPU 70 (**a**, **d**), TPU 80 (**b**, **e**) and TPU 95 (**c**, **f**)

Three different TPUs (95A, 80, 70) with differing degrees of elasticity were tested at first considering their elasticity, flexibility and printability. Printing process speed has been tested starting from 5.0 mm s^−1^ for TPU 95 to 1.0 or 2.0 mm s^−1^ for TPU 80 and 70, respectively. Low speed is required due to sticky material characteristics and shape instability. Nozzle moving with high speed generally deposited excess molten TPU causing shape instability and losing some material, which could result in the presence of holes in the final graft. Nozzle travel speed from side to side was considered too; high speed during process could move the deposited graft on the stage causing possible holes to be present at the end of printing. Surface morphology of different printed vascular grafts was then studied with SEM (Fig. 3) to evaluate which filament created smooth and linear surfaces. Light microscopy was also used to evaluate graft size and wall thickness. From SEM analysis, 3D printed vascular grafts showed a linear surface. Light microscopy showed good outline scaffold and accurate wall thickness. However, TPU 80 showed smoother surface. TPU 70 was almost immediately discarded from the study due to difficulty in printing of this material. Consequently, due to TPU 70 strong sticky characteristics, Bowden tube needed to be lubricated using glycerine to avoid it getting stuck during printing process.

**Fig. 4:**
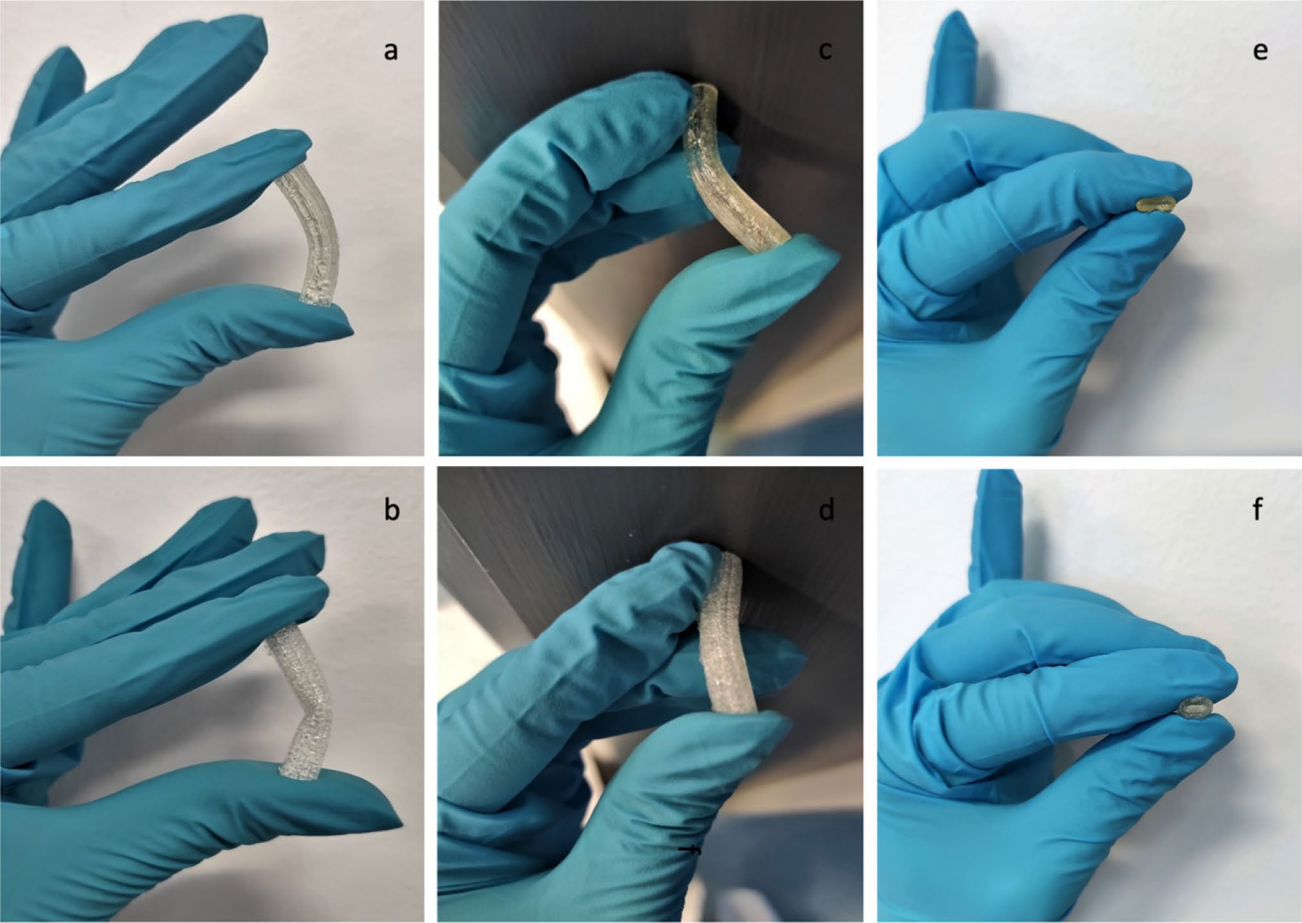
Elasticity and strength of vascular grafts printed with TPU 80 (**a**, **c**, **e**) and TPU 95 (**b**, **d**, **f**)

TPU 80 was chosen for this work consequently to its favoured physical characteristics of elasticity and flexibility, compared to TPU 95 (Fig. 4). TPU 80 has a better printability compared to TPU 70; printing process parameters have been adjusted as listed in Table 2 for each material. As listed in Table 2, 1 mm s^−1^ speed was chosen to print TPU 80, as well as layer height which was decreased to the lowest value of 0.1 mm; consequently, lower values will produce slower prints in high resolution. Initial layer height has also been set to 0.1 mm and line width to 0.3 mm. Infill density has been settled at 100% to obtain regular surface, avoiding the presence of holes. Bowden lubricated tube had to be used during printing process due to sticky characteristics.

**Fig. 5:**
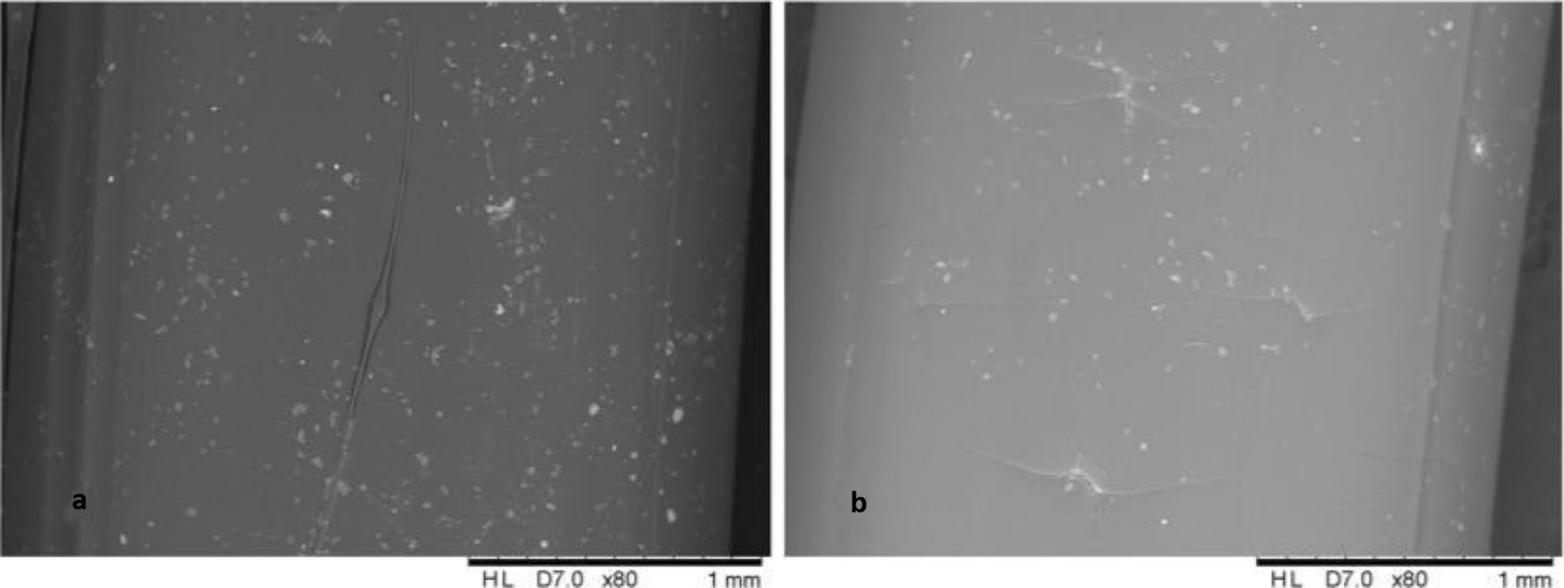
SEM images of TPU filaments containing curcumin (**a**) and placebo TPU 80 filaments (**b**)

**Fig. 6:**
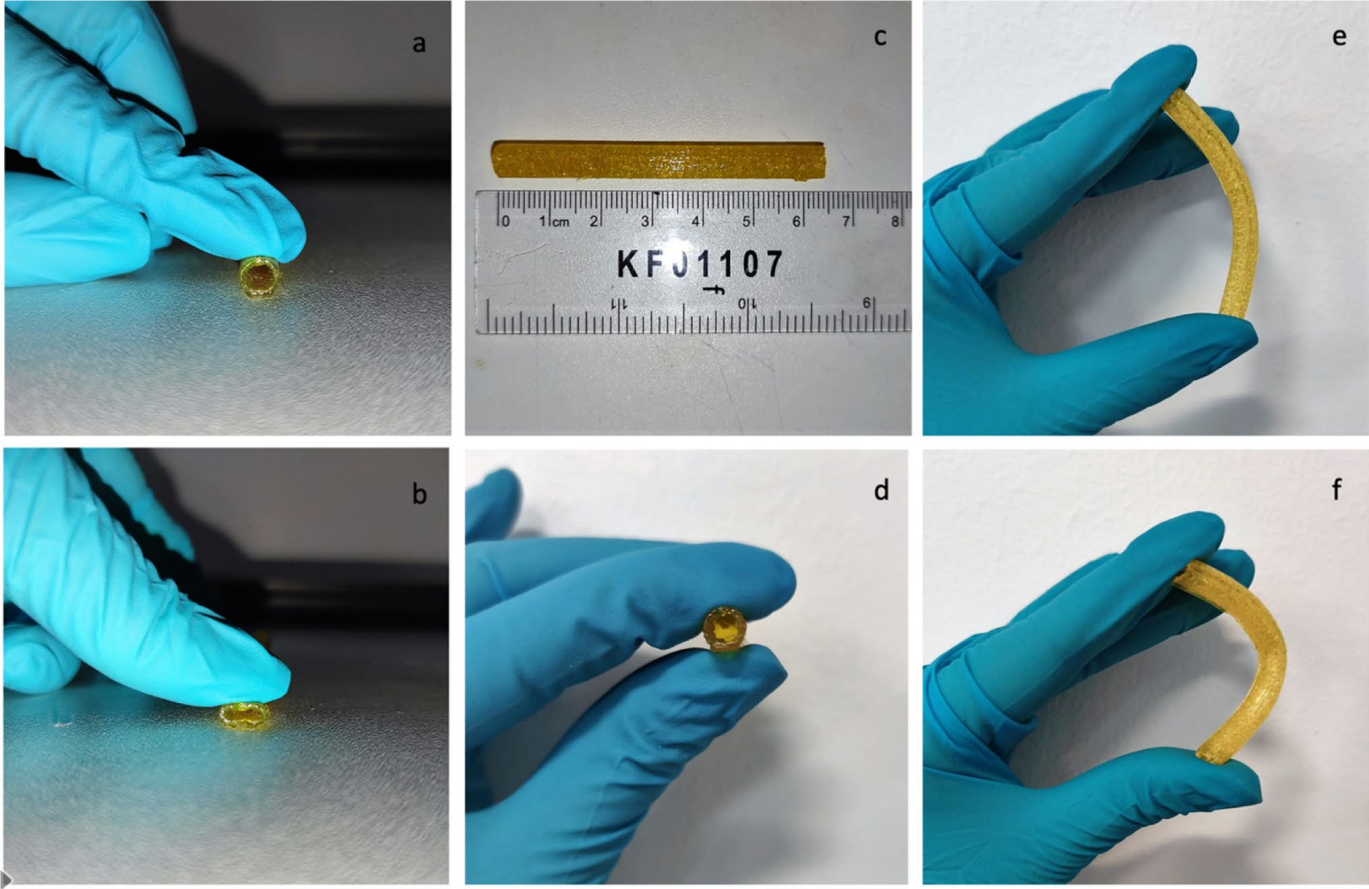
3D printed curcumin-loaded vascular grafts

Smooth filaments of 2.85 mm in diameter were produced through HME using TPU 80 pellets and 0.25% curcumin powder. Curcumin was mixed successfully with TPU matrix, as SEM analysis (Fig. 5) confirmed the homogenous distribution of curcumin along the filament with no aggregates within the extruded filament. This can be also inferred by filament colour (Fig. 6) where a homogenous colour is present throughout the tube.

**Fig. 7:**
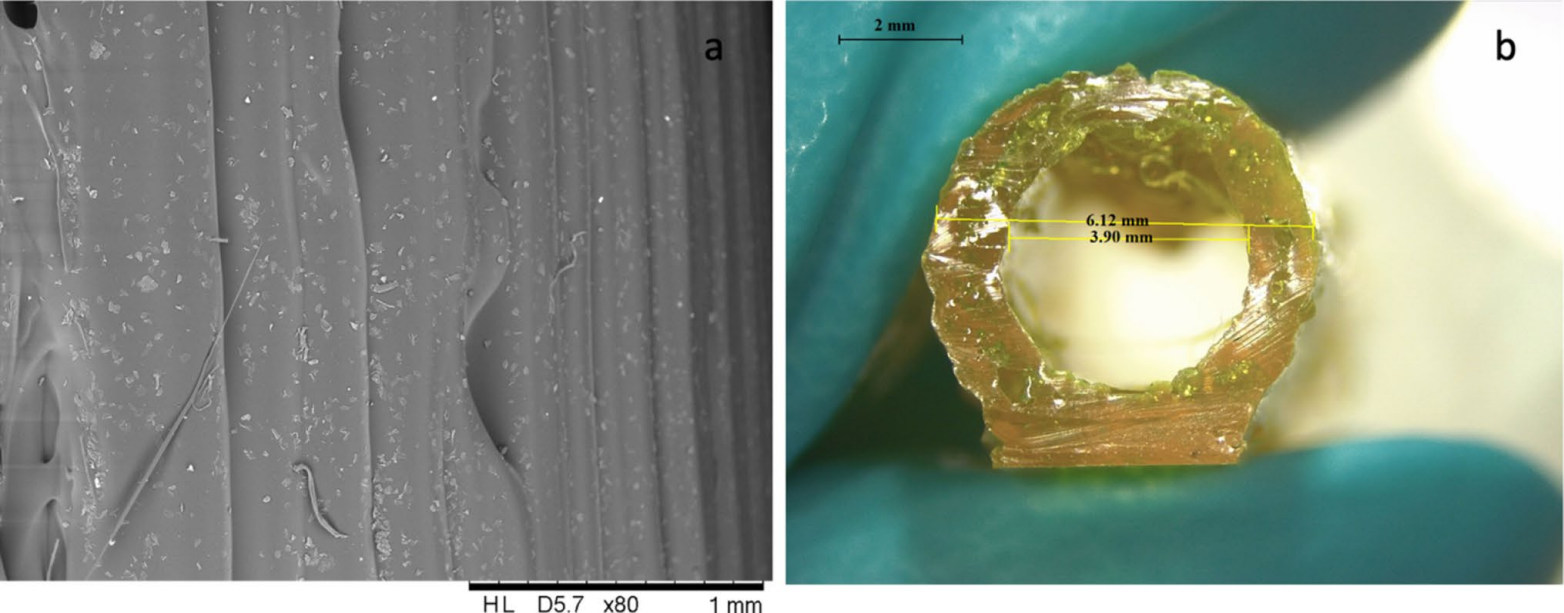
3D printed curcumin loaded vascular grafts: SEM image (**a**) and lighting microscopy images (**b**)

Vascular grafts were printed and characterized using the curcumin loaded extruded filaments with straight shape of 65 mm length, OD of 6.0 mm and ID of 4.0 mm (Figs. 6 and 7), using the same adjusted printing process parameters used for TPU 80, demonstrating no changes due to the presence of curcumin on graft printability.

### FT-IR characterization

**Fig. 8:**
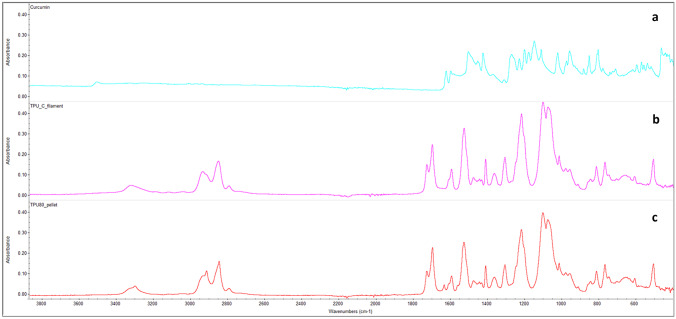
FT-IR spectra of curcumin powder (**a**), TPU filaments containing curcumin (**b**) and TPU 80 pellet (**c**)

FT-IR spectra (Fig. 8) were obtained from curcumin powder, TPU filaments containing curcumin and TPU 80 pellet, to establish possible interaction between different materials. No interaction between curcumin and TPU were shown, probably due to the lower concentration of curcumin used for this study.

### Thermal analysis

**Fig. 9:**
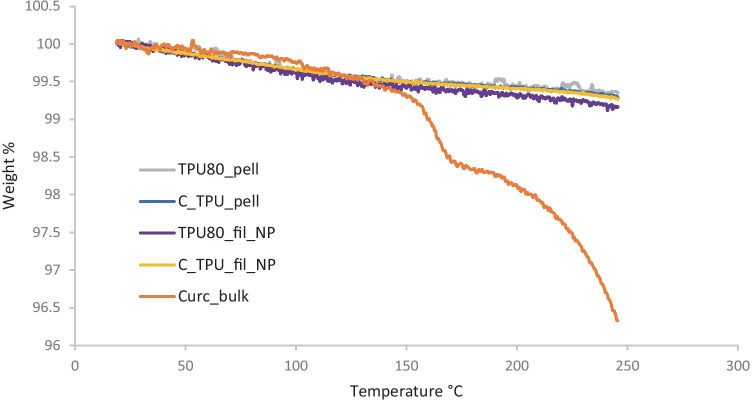
TGA graphs of bulk curcumin, thermoplastic polyurethane (TPU) 80 filament (Fil) not printed (NP), TPU filaments containing curcumin (C) not printed, TPU 80 and TPU 80 pellets containing curcumin

**Fig. 10:**
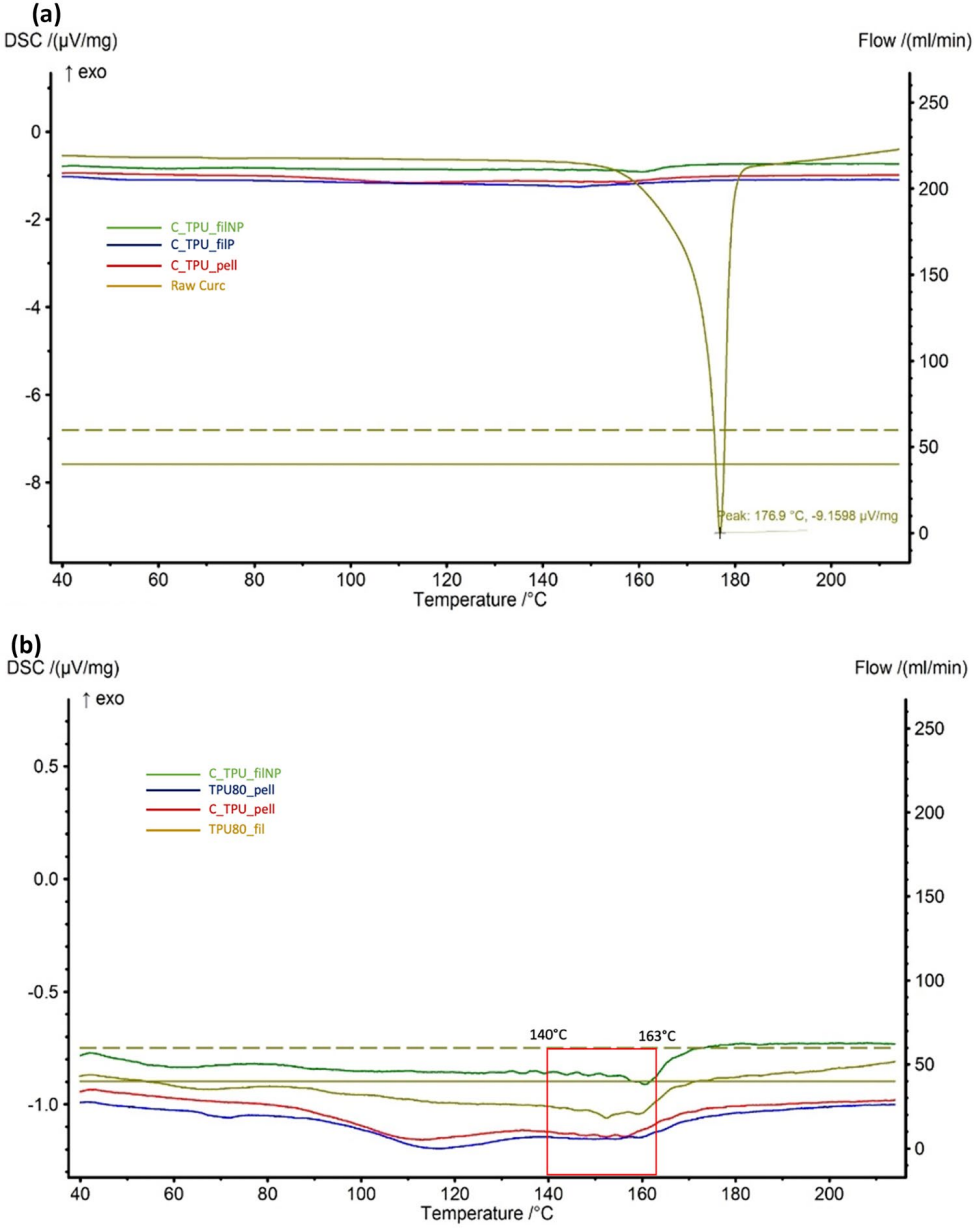
DSC graphs of TPU 80 filament containing curcumin not printed and printed, TPU 80 pellet containing curcumin and raw curcumin (the entire graphs can been seen in (**a**), and under (**b**), the specific areas have been zoom-in). Green line represents C-TPU-filNP, blue line TPU80_pell, red line C_TPU_pell and orange line TPU80_fil

TGA (Fig. 9) and DSC (Fig. 10) analyses were conducted on three samples of curcumin powder, TPU 80 pellet and filament not printed (NP), TPU filaments containing curcumin pellet NP, in a range temperature between 25 and 250 °C, to investigate also the HME (200 °C at maximum) and printer (210 °C nozzle temperature) temperatures.

Thermal analysis also did not show any curcumin influence in filament stability, considering TPU has maintained a good stability at high temperature, despite curcumin instability. Curcumin started degrading around 180 °C, according to its melting point [30]; otherwise, no peaks associated with curcumin degradation could be noticed in filament containing curcumin and pellet. However, neat TPU naturally include hard segment and soft segment with two different melting temperatures [31]. Soft segment peak usually around 20 °C could not be noticed in the analysis because 25 °C was used as initial temperature; hard segments melting temperature could be noticed in Fig. 9, around 110 °C in TPU 80 pellet and TPU pellet containing curcumin [32]. However, thermal analysis demonstrated TPU stability at high temperature used for extrusion and printing.

### Degradation studies

**Fig. 11:**
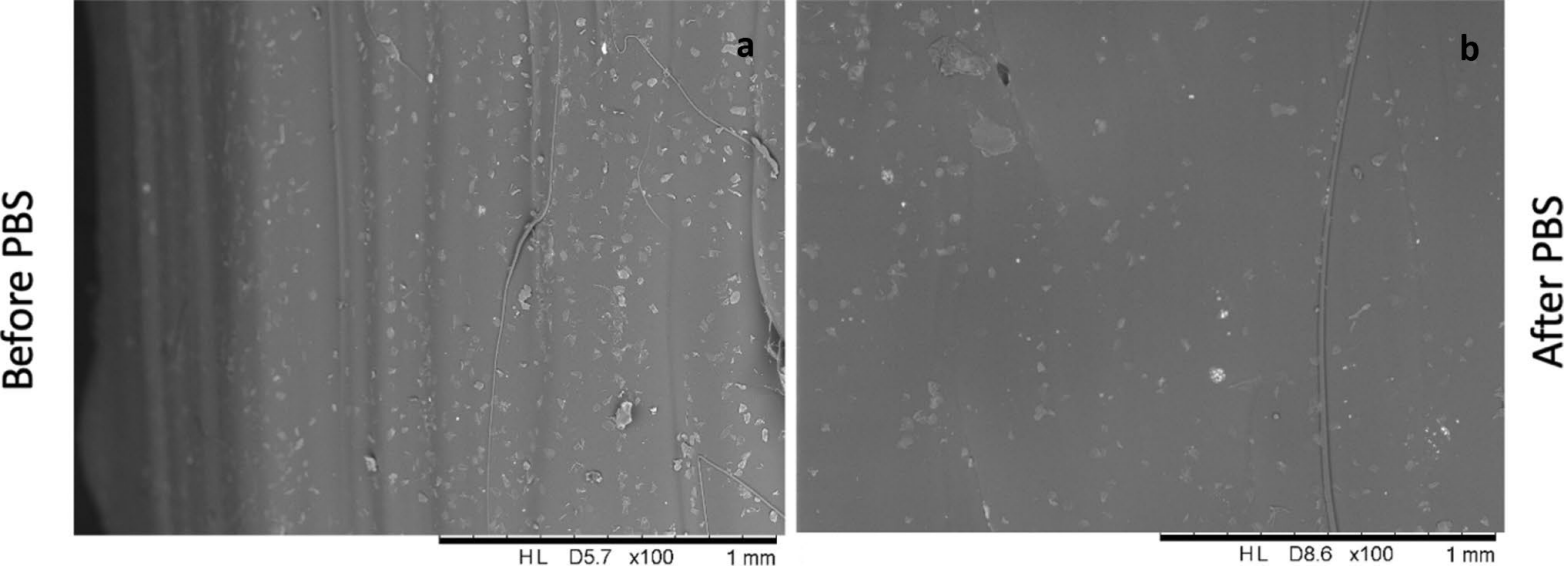
Curcumin loaded vascular grafts before (**a**) and after (**b**) placement in PBS solution for 3 weeks

Degradation studies were performed by placing grafts containing curcumin into PBS, to analyse surface modifications after 3 weeks (Fig. 11). The graft weight was also considered, with differences remaining within statistical accepted values starting from 156.3 mg at time 0 to 160.9 mg on 3rd week.

#### Mechanical properties of vascular grafts

**Fig. 12:**
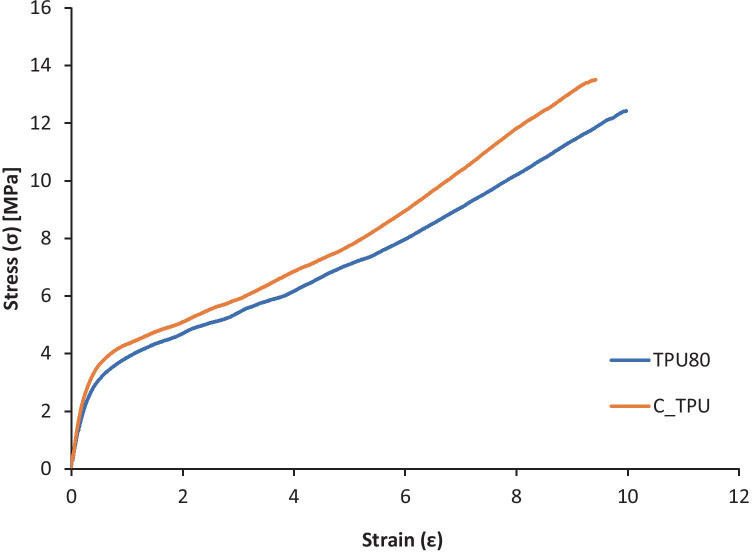
Stress and strain graphs of TPU filament containing curcumin and TPU placebo filament

TPU 80 and TPU containing curcumin were evaluated to demonstrate no changes in mechanical properties after curcumin loading. The filament containing curcumin samples did not break even after 20 cm distance, which was the maximum distance input into the texture analyser. Same results were also obtained with TPU 80 samples. Ultimate tensile strength (UTS), maximum elongation, and elastic modulus obtained from stress and strain curve (Fig. 12) and stiffness (Table 4) were calculated for each sample (*n* = 3).

**Table 4:** UTS, maximum elongation, elastic modulus, and stiffness for TPU 80 placebo and curcumin-loaded filaments

**Filament**	**UTS (Mpa)**	**Max elongation (%)**	**Elastic modulus (Mpa)**	**Stiffness (N mm**^−**1**^**)**
**Curcumin-loaded TPU 80**	11.65 ± 1.68	9.16 ± 0.24	10.56 ± 2.19	2.61 ± 0.46
***Placebo TPU 80***	13.41 ± 1.43	9.97 ± 0.02	9.02 ± 0.54	2.22 ± 0.14

As shown in Table 4, clear differences could be noticed between curcumin-loaded TPU and TPU 80 UTS, while there are no changes with max elongation. However, the extruded filament reserves good elasticity considering no breaks were noticed during analysis of all three samples.

### In vitro release of curcumin from vascular grafts

**Fig. 13:**
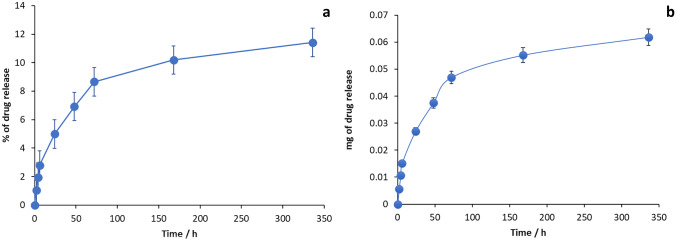
Cumulative percentage of drug release (**a**) and cumulative mg of drug release (**b**)

Drug release studies were analysed using UV analysis at 425 nm length. Due to curcumin’s low solubility, vascular grafts were placed in 3 mL of PBS solution containing 0.5% v/v Tween 80. It is well known that curcumin exhibits a pH-dependent stability in acidic conditions; in vitro release study was carried out at acidic pH (pH 6.0) [33]. After several literature studies, Tween 80 turned out to be a good additive into buffer, which has been used in different studies in order to sustain curcumin release [26–28]. Several manuscripts have also added methanol into buffer, in order to have a certain curcumin release, even if methanol is not assessed in physiological conditions [34, 35]. An initial burst release was detected at day 1, and the release gradually continued until the end of the 2-week study (Fig. 13). Curcumin released was around 61 μg compared to 75 mg used for filament extrusion. Considered the small loss of curcumin during HME, around 80% was incorporated into grafts; as previously reported [17, 36], curcumin might be interacting with TPU due to TPU hydrophobic nature, limiting drug release; therefore, curcumin will continue to release also after the 2 weeks. Using material such as TPU, drug could remain in the graft for a longer period if grafts remain permanently in the patient body. However, considering curcumin activity, the amount of curcumin required to exhibit anticoagulant effects is around 5.5 mg to match heparin activity. Seventy-five milligrams of curcumin was used to prepare filament; in this way, around 10.83 mg of curcumin could be enough to be used in a 20-cm vascular graft. Low curcumin concentration (0.25%) was chosen at first to demonstrate printability of drug within main material. In order to have a higher release concentration, curcumin amount in filament should be increased. The curcumin amount which could be released from 20 cm graft might be capable in exhibiting an anti-bacterial effect; curcumin MIC is between 129 and 293 μg mL^−1^ [25], 10.83 mg is present in 20 cm vascular graft as cited before and 411.35 μg mL^−1^ (12%) will be released in 2 weeks.

## Conclusions

This study demonstrates the potential use of 3DP FDM method in the manufacture of vascular grafts, in order to overcome inter-personal characteristics and obtain personalized grafts suitable for different vein and arterial sizes. User-friendly CAD software and speedy printing process allow personalized vascular graft production. Different shaped designs could overcome kinking problems caused by straight shape in commerce. Thanks to 3DP technique, in several years, it could be possible to manufacture directly patient veins and arteries to create a personalized graft in a short time, avoiding failing maturation problem and infection. 3DP method was validated using a FDM printing technique despite difficult cylindrical configuration to obtain. Moreover, incorporation of drugs in TPU using HME has been demonstrated with SEM and TGA. It has also been shown that the presence of drug does not influence TPU mechanical properties. Filaments obtained were successfully used to prepare vascular grafts using 3DP. Anti-coagulant drug incorporation could avoid thrombosis problem generally caused using grafts.

The methods, described in this manuscript, could be used for different medical applications, as well as ease incorporation of drugs. One-step filament production could lead to short time production process and low costs; drug melting point and temperature degradation have to be investigated in order to demonstrate the possibility of incorporation.

## Data Availability

The datasets generated during and/or analysed during the current study are available from the corresponding author on reasonable request.
